# Long non-coding RNA LINC00511 facilitates colon cancer development through regulating microRNA-625-5p to target WEE1

**DOI:** 10.1038/s41420-021-00790-9

**Published:** 2022-04-27

**Authors:** Xiaowu Qian, Chun Jiang, Zhengtai Zhu, Gaohua Han, Ning Xu, Jun Ye, Ruixing Wang

**Affiliations:** 1 Department of Geriatrics, Taizhou People’s Hospital (Taizhou People’s Hospital affiliated to Nanjing Medical University), 225300 Taizhou, Jiangsu China; 2 Department of Cardiology, Taizhou People’s Hospital (Taizhou People’s Hospital affiliated to Nanjing Medical University), 225300 Taizhou, Jiangsu China; 3 Department of Oncology, Taizhou People’s Hospital (Taizhou People’s Hospital affiliated to Nanjing Medical University), 225300 Taizhou, Jiangsu China; 4 Department of Gastrointestinal Surgery, Taizhou People’s Hospital (Taizhou People’s Hospital affiliated to Nanjing Medical University), 225300 Taizhou, Jiangsu China; 5 Department of Central Laboratory, Taizhou People’s Hospital (Taizhou People’s Hospital affiliated to Nanjing Medical University), Taizhou, 225300 Jiangsu China

**Keywords:** Cancer, Diseases

## Abstract

The altered part of long non-coding RNA LINC00511 (LINC00511) is extensively discussed in malignancies. Finitely, the mechanism of LINC00511 in colon cancer (CC) development lacks thorough explorations. Hence, this work is started from the LINC00511-mediated microRNA (miR)-625-5p/WEE1 axis in the CC process. LINC00511, miR-625-5p, and WEE1 levels were tested in CC tissues and cells. Subcellular localization of LINC00511 was clarified. CC cells were transfected with oligonucleotides that altered LINC00511, and miR-625-5p expression to define their performance in CC cell progression. The tumorigenic ability of cells was verified in xenografted tumors. CC tissues and cells highly expressed LINC00511 and WEE1 and lowly expressed miR-625-5p. LINC00511 was mainly localized in the cytoplasm. Deleted LINC00511 or restored miR-625-5p delayed cellular growth in CC. LINC00511 sponged miR-625-5p to target WEE1. Silenced miR-625-5p mitigated the role of depleted LINC00511, while inhibited WEE1 rescued the effect of silenced miR-625-5p on the biological functions of CC cells. It is summarized that down-regulated LINC00511 obstructs tumorigenesis of CC through restoring miR-625-5p and silencing WEE1, consolidating a basal reference for CC-oriented therapy.

## Introduction

Colon cancer (CC) registers as the third most widespread cancer, with high occurrence and mortality rates [1]. According to anatomical positions, CC is divided into two subtypes, right CC and left CC, showing different molecular characteristics and discrepant strategies [2]. Screening colonoscopy is effectively applied in the diagnosis of CC due to its benefits in reducing death rate and survival duration [3]. In addition, surgical resection of the colon is a well-established therapy for CC, while adjuvant chemotherapy followed by surgery further reduces the recurrence rate of CC [4]. As to the high prevalence, it is critical to explore optimal agents to minimize the impacts induced by CC.

The cooperation of long non-coding RNAs (lncRNAs) and microRNAs (miRNAs) has been implied in the molecular movement of CC. For example, lncRNA metastasis-associated lung adenocarcinoma transcript 1 serves in the regulation of biological behaviors of CC cells through interfering with miR-21 [5]. Additionally, lncRNA differentiation antagonizing non-protein coding RNA has been witnessed to promote cellular growth and metastasis in CC through targeting miR-518a-3p [6]. According to former research, it is illustrated that abnormally expressed lncRNA LINC00511 (LINC00511) functions in the biological behaviors of colorectal cancer (CRC) [7]. Pertaining to the miRNA collection, miR-625 is implied to relate to microsatellite instability in CC [8] and is differently expressed in healthy controls and colorectal adenomas [9]. Creatively, miR-625-3p is hinted to negatively connect with the outcomes of first-line oxaliplatin-based chemotherapy in metastatic CRC [10] and down-regulated miR-625-5p is responsible for accelerating CRC cell progression [11]. Though the ceRNA network of LINC00511 and miR-625 has been explored in tumor cell progression [12], the synergism of LINC00511 and miR-625-5p has been rarely discussed in CC. WEE1, defined as an oncogenic G_2_ checkpoint kinase, works in the field of modulating the proliferation of CC cells [13]. Ge et al. have surveyed that WEE1 is substantially connected with distant metastasis and advanced tumor node metastasis stage in CRC [14]. Also, it is manifested that depletion of WEE1 is conducive to the suppressed proliferation ability of CRC cells [15]. Illuminated by these researches, our research was initiated to explore the function of LINC00511, miR-625-5p, and WEE1 in the malignant phenotype of CC.

## Results

### LINC00511 attains a high level while miR-625-5p a low level in CC

**Fig. 1:**
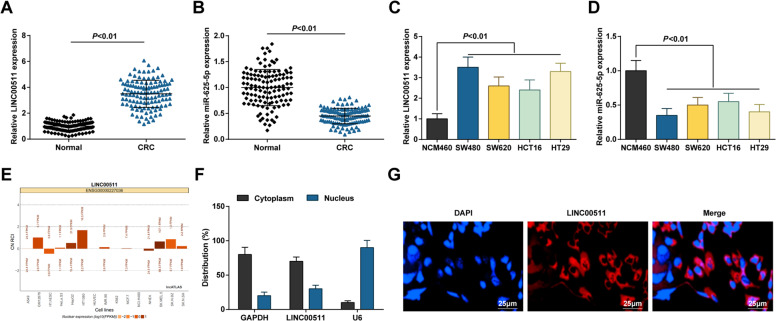
LINC00511 attains a high level while miR-625-5p a low level in CC. **A** RT-qPCR detected LINC00511 expression in CC tumor tissues and normal tissues (*n* = 120); **B** RT-qPCR detected miR-625-5p expression in CC tumor tissues and normal tissues (*n* = 120); **C** RT-qPCR detected LINC00511 expression in CC cell lines and normal epithelial cells; **D** RT-qPCR detected miR-625-5p expression in CC cell lines and normal epithelial cells; **E** Bioinformatics website predicted the subcellular localization of LINC00511; **F** subcellular separation assay detected subcellular localization of LINC00511; **G** FISH assay detected subcellular localization of LINC00511; the measurement data are expressed as mean ± standard deviation. *N* = 3.

It has been proved that LINC00511 promoted the proliferation of CRC and its immune activity was enhanced in CRC tissues [7]. In addition, the anti-tumor effect of miR-625-5p has been evidenced in cancers [16, 17]. Based on these facts, the functions of LINC00511 and miR-625-5p in CC have been first clarified through detecting LINC00511 and miR-625-5p expression in CC tumor tissues and normal tissues, as well as CC cell lines and human normal intestinal epithelial cells NCM460. Reverse transcription-quantitative polymerase chain reaction (RT-qPCR) clearly manifested that LINC00511 was up-regulated and miR-625-5p was down-regulated in CC tumor tissues (Fig. 1A, B) and CC cell lines (Fig. 1C, D). The lncATLAS website predicted the subcellular localization of LINC00511 while subcellular separation and fluorescence in situ hybridization (FISH) assays found that LINC00511 was mainly located in the cytoplasm (Fig. 1E–G).

### LINC00511 knockdown destroys CC cell viability and colony forming ability and encourages apoptosis

**Fig. 2:**
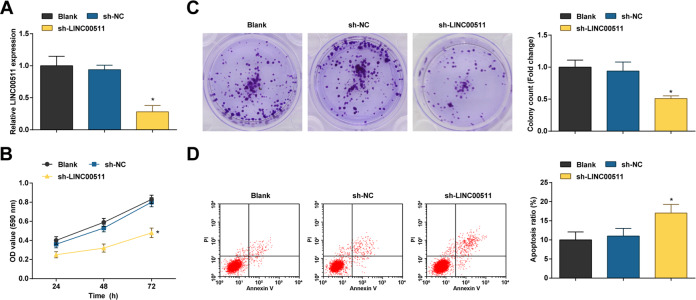
LINC00511 knockdown destroys CC cell viability and colony forming ability and encourages apoptosis. **A** RT-qPCR detected LINC00511 expression in CC cells; **B** MTT assay detected viability of CC cells; C colony formation assay detected colony-forming ability of CC cells; D Flow cytometry detected apoptosis rate of CC cells; the measurement data are expressed as mean ± standard deviation. *N* = 3. *P < 0.05 compared with the sh-NC group.

The functional relevance of dysregulated LINC00511 in CC was investigated in SW480 cells. Upon sh-LINC00511 having been introduced into SW480 cells, LINC00511 expression was knocked down in SW480 cells (Fig. 2A).

Detected by 3-(4, 5-dimethylthiazol-2-yl)-2, 5-diphenyltetrazolium bromide (MTT) assay, colony formation assay, and flow cytometry, it was displayed that knocking down LINC00511 impaired cell viability and the formation of cell colonies and accelerated apoptosis of SW480 cells (Fig. 2B–D).

### LINC00511 knockdown impairs the migration and invasion of CC cells and cellular growth in mice

**Fig. 3:**
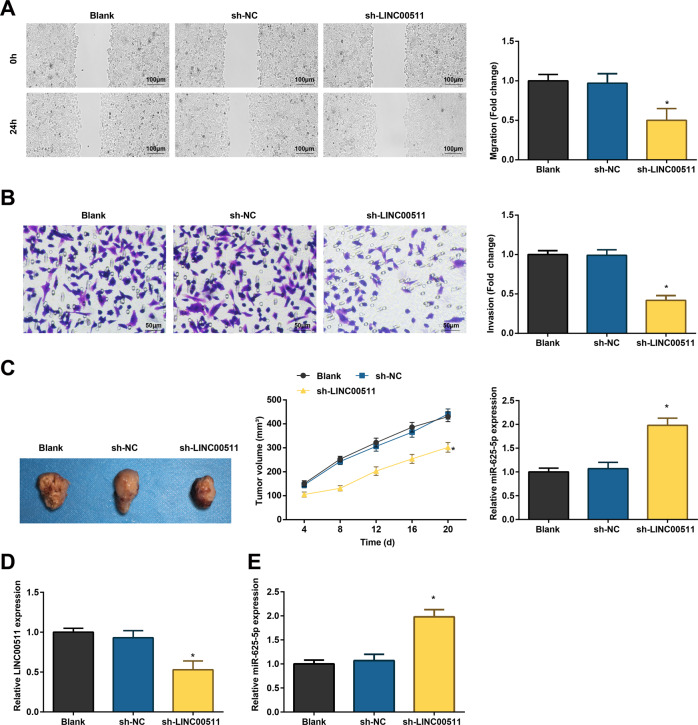
LINC00511 knockdown impairs CC cell migration and invasion and cellular growth in mice. **A** Scratch test detected migration ability of CC cells; **B** Transwell assay detected invasion ability of CC cells; **C** xenografted tumor volume and weight; **D** RT-qPCR detected LINC00511 expression in tumors; **E** RT-qPCR detected miR-625-5p expression in tumors; the measurement data are expressed as mean ± standard deviation. *N* = 3. *P < 0.05 compared with the sh-NC group.

Through scratch test and Transwell assay, it was revealed that depleting LINC00511 lowered the migration and invasion rate of SW480 cells (Fig. 3A, B). LINC00511-regulated tumorigenesis of CC in vivo was tested by subcutaneously injecting SW480 cells (transfected with sh-LINC00511 and sh-NC) into the dorsal side of nude mice. Smaller and lighter tumors were seen in mice injected with depleted LINC00511 (Fig. 3C). In addition, reduced LINC00511 and increased miR-625-5p levels were recognizable in mice injected with sh-LINC00511-transfected SW480 cells (Fig. 3D, E).

### LINC00511 negatively targets miR-625-5p

**Fig. 4:**
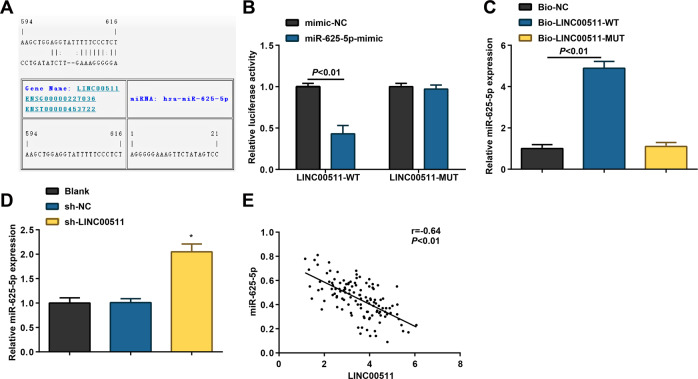
LINC00511 negatively targets miR-625-5p. **A** RNA22 predicted the binding site of LINC00511 and miR-625-5p; **B** dual-luciferase reporter gene assay detected the targeting relationship between LINC00511 and miR-625-5p; **C** RNA pull-down assay verified the targeting relationship between LINC00511 and miR-625-5p; **D** RT-qPCR detected miR-625-5p expression after knockdown of LINC00511; **E** Pearson correlation analysis evaluated the correlation between LINC00511 and miR-625-5p in CC tumor tissues (*n* = 120); the measurement data are expressed as mean ± standard deviation. *N* = 3. *P < 0.05 compared with the sh-NC group.

In the xenografted tumors, miR-625-5p expression was elevated upon LINC00511 down-regulation. To define the action between LINC00511 and miR-625-5p, the RNA22 website was applied to perform binding site analysis between LINC00511 and miR-625-5p (Fig. 4A), which was followed by dual-luciferase reporter gene and RNA pull-down assays to confirm that LINC00511 was bound to miR-625-5p (Fig. 4B, C).

Besides that, miR-625-5p expression after LINC00511 down-regulation was tested to increase in SW480 cells (Fig. 4D). Moreover, miR-625-5p and LINC00511 were negatively correlated in CC tissues (Fig. 4E), supporting that LINC00511 negatively regulated miR-625-5p expression.

### Restored miR-625-5p destroys CC cell viability and colony forming ability and encourages apoptosis

**Fig. 5:**
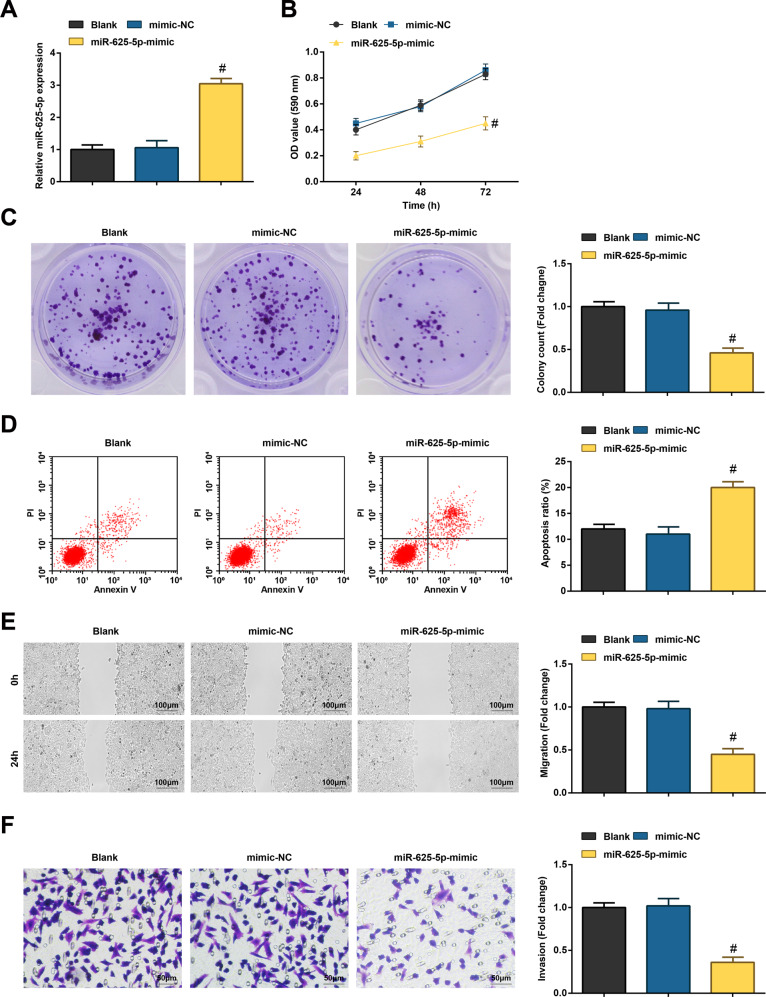
Restored miR-625-5p destroys CC cell viability and colony forming ability and encourages apoptosis. **A** RT-qPCR detected miR-625-5p expression in CC cells; **B** MTT assay detected viability of CC cells; **C** colony formation assay detected colony-forming ability of CC cells; **D** flow cytometry detected apoptosis rate of CC cells; **E** scratch test detected migration ability of CC cells; **F** Transwell assay detected invasion ability of CC cells; the measurement data are expressed as mean ± standard deviation. *N* = 3. ^#^*P* < 0.05 compared with the mimic-NC group.

miR-625-5p-regulated SW480 cells were established by transfecting with miR-625-5p mimic (Fig. 5A). Various assays discovered that restoring miR-625-5p would result in damages in cell viability, colony-forming, migration, and invasion capacities, along with induction of apoptosis (Fig. 5B–F).

### LINC00511 positively while miR-625-5p negatively connects with WEE1

**Fig. 6:**
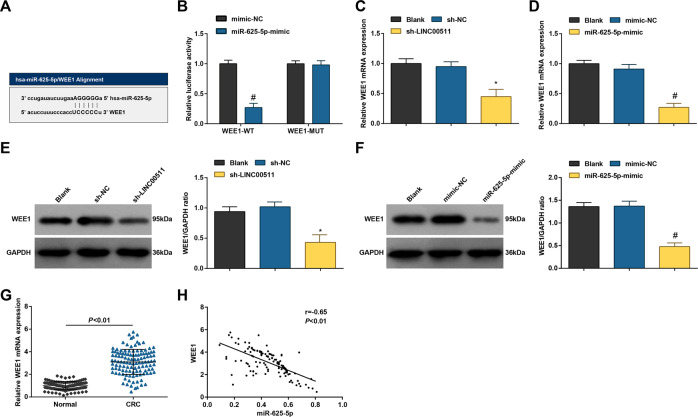
LINC00511 positively while miR-625-5p negatively connects with WEE1. **A** StarBase website predicted the binding site of miR-625-5p and WEE1; **B** dual-luciferase reporter gene assay analyzed the binding site of miR-625-5p and WEE1; **C** RT-qPCR detected WEE1 mRNA expression in CC cells after regulation of LINC00511 and miR-625-5p; **D** western blot assay detected the WEE1 protein expression in CC cells after regulation of miR-625-5p; **E**, **F** western blot assay detected WEE1 protein expression in CC cells after regulation of LINC00511 or miR-625-3p; **G** RT-qPCR detected the WEE1 expression in CC tumor tissues and normal tissues (*n* = 120); **H** Pearson correlation analysis evaluated the correlation between WEE1 and miR-625-5p in CC tumor tissues (*n* = 120); The measurement data are expressed as mean ± standard deviation. *N* = 3. ^#^*P* < 0.05 compared with the mimic NC group. *P < 0.05 compared with the sh-NC group.

WEE1 was reported to be overexpressed in CRC clinical samples [14]. As manifested in Fig. 6A, B, miR-625-5p could bind to the 3’-untranslated region of WEE1 and regulate WEE1 expression in SW480 cells. Also, RT-qPCR and Western blot assay illustrated that reducing LINC00511 or elevating miR-625-5p abated WEE1 expression in SW480 cells (Fig. 6C–F). Consistently, WEE1 expression was enriched in CC tissues (Fig. 6G), and it was negatively connected with miR-625-5p expression (Fig. 6H).

### Silenced miR-625-5p reverses depleted LINC00511-induced effects on CC cells

**Fig. 7:**
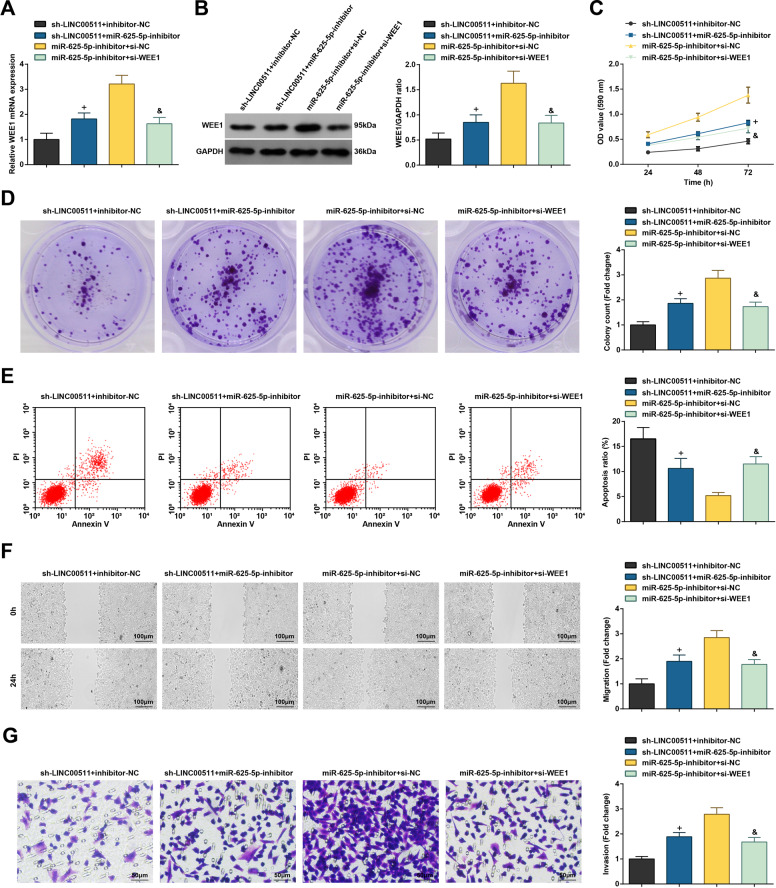
Silenced miR-625-5p reverses depleted LINC00511-induced effects on CC cells. **A** RT-qPCR detects WEE1 mRNA expression in CC cells; **B** western blot assay detected WEE1 protein expression in CC cells; **C** MTT assay detected viability of CC cells; **D** colony formation assay detected colony-forming ability of CC cells; **E** flow cytometry detected apoptosis rate of CC cells; **F**. Scratch test detected migration ability of CC cells; **G**. Transwell assay detected invasion ability of CC cells; the measurement data are expressed as mean ± standard deviation. *N* = 3. ^+^*P* < 0.05 compared with the sh-LINC00511 + inhibitor-NC group; ^&^*P* < 0.05 compared with the miR-625-5p-inhibitor + si-NC group.

The carcinogenic effect of miR-625-5p/WEE1 axis-mediated LINC00511 on CC was decoded. sh-LINC00511 + inhibitor-NC, sh-LINC00511 + miR-625-5p-inhibitor, miR-625-5p-inhibitor + si-NC, and miR-625-5p-inhibitor + si-WEE1 were transfected into SW480 cells, respectively. RT-qPCR and western blot analysis proved the successful transfection (Fig. 7A, B). Next, in SW480 cells, it was observed that the biological progress of SW480 cells suppressed by sh-LINC00511 was reversed by miR-625-5p-inhibitor transfection. Also, si-WEE1 could antagonize the tumor role of miR-625-5p-inhibitor in SW480 cells (Fig. 7C–G).

## Discussion

Established as the common malignant tumor, CC is often accompanied by higher mortality and morbidity [18]. The lncRNA-miRNA-mRNA network has been constructed in CC. On the basis of that, this work is guided toward decoding the mechanism of the LINC00511-miR-625-5p-WEE1 network in the biological behaviors of CC cells. Ultimately, the research works out that silencing LINC00511 up-regulated miR-625-5p to restrain WEE1, thereafter to block the way of CC cell progression.

First, LINC00511 expression was detected in CC tissues and cells for further explorations of its movements in CC. By so doing, up-regulated LINC00511 was exhibited in CC. To proceed, LINC00511 down-regulation assays were implemented on SW480 cells to convince the suppressive functions of depleted LINC00511 in cell viability, colony-forming, migration, and invasion abilities and the promoting functions in apoptosis. Consistently, the aggressive growth of xenografted tumors in mice was suppressed by depleted LINC00511. To support the result in this work, current work has elucidated that LINC00511 is overexpressed in CRC, contributing to augmenting proliferation and impeding apoptosis of CRC cells, whereas depleted LINC00511 delays tumorigenesis in animal models [7]. Except for CC, the promoting actions of LINC00511 have been reflected in other cancers. Apparently, the overexpressed LINC00511 is manifested in gastric cancer, and LINC00511 knockdown encumbers cell maturity and increases cell death ratio [19, 20]. Likewise, LINC00511 expression attains a high level in esophageal cancer, and further enhancement of LINC00511 accelerates the process of proliferation and migration and hinders cell apoptosis [21]. In a similar fashion, highly expressed LINC00511 in cervical cancer is inhibited by si-LINC00511, thereby repressing cellular proliferation and encouraging apoptosis, and destroying tumorigenesis in mice [22]. Compatibly, LINC00511 expression goes toward an increase in papillary thyroid carcinoma, which crucially excites G_1_/S transition and cell progression vitality [23]. Anyhow, the actions of LINC00511 in impelling the malignant development of other cancers are echoed with those in CC.

Afterward, the potential relationship between LINC00511 and miR-625-5p was predicted and validated, as reflected by the fact that LINC00511 is bound to miR-625-5p. As to miR-625-5p, its expression was down-regulated in CC, and functionally, its restoration contributed to the restricted cellular growth. However, inhibited miR-625-5p suppressed the anti-tumor role of depleted LINC00511 on CC cells. In fact, binding sites do exist between miR-625-5p and LINC00511, further proving the negative relation between those two actors [24]. With prognostic meaningfulness, miR-625 is reported to be down-regulated in CRC [25]. In an experiment conducted by Shang T et al., the kenspeckle decrease is noticed in miR-625-5p expression in CRC and the down-regulated miR-625-5p triggers CRC cells to perform aggressively [11]. In a similar way, depressed miR-625 expression is manifested in CRC while the miR-625 restoration is devoted to impairing the abilities of tumor cells to invade and migrate in vitro and in vivo [26]. Moreover, miR-625-5p expression is intended to reduce lung adenocarcinoma and its down-regulation stimulates malignant cells to act in an aggressive way [27].

Subsequently, the substantial link between miR-625-5p and WEE1 showed that miR-625-5p was negatively correlated while LINC00511 was positively correlated with WEE1 expression in CC. Our study found the up-regulated WEE1 in CC and further proved that silenced WEE1 rescued the pro-tumor effect of inhibited miR-625-5p on CC. WEE1 mRNA expression is inclined to elevate in CRC, dramatically connecting with CRC metastasis [14]. Drawn from observational works, WEE1 depletion attacks the proliferative ability of CC cells [13] and has the potency to reverse G_2_/M cell cycle checkpoint activation in cancers [28]. From a wide perspective, spontaneous inhibition of WEE1 and Chk1 takes over premature mitosis before DNA replication, causing apoptosis and disturbed tumor growth [29]. Anyway, the above researches all support the positive behaviors of depleted WEE1 in cancers.

All in all, it is explanatory that LINC00511 initiates and expands the carcinogenic growth of CC cells through repressing miR-625-5p and enhancing WEE1. Due to the limitations in the relatively small scale of this work, much more researches are required to explore LINC00511/miR-625-5p/WEE1 axis on a larger scale.

## Methods and materials

### Clinical specimens

A total of 120 cases of CC tissue and normal tissues specimens were resected from CC patients. All specimens were confirmed in the Department of Pathology, and relevant clinical data were collected. Patients having received radiotherapy or chemotherapy before surgery were excluded. The specimens were frozen in liquid nitrogen and preserved at −80 °C [30].

### Cell culture

Human CC cells SW480, SW620, HCT16, and HT29 and normal human colon mucosal epithelial cell line NCM460 (ATCC, VA, USA) were obtained from ATCC (Manassas, VA, USA) and cultured as per their instructions. All cells were incubated following their instructions at 37 °C with 5% CO_2_ [31].

### Cell transfection

Cells of passage 3 were trypsinized and cultured in 24-well plates (2 × 10^6^ cells/well) to grow into a single layer. Then, cells were transfected with negative control short hairpin RNA (sh-NC), LINC00511 shRNA (sh-LINC00511), mimic NC, miR-625-5p mimic, sh-LINC00511  + miR-625-5p-inhibitor NC, sh-LINC00511 + miR-625-5p inhibitor, miR-625-5p-inhibitor + si-NC, or miR-625-5p-inhibitor + si-WEE1 by LiPofectamine 2000 following the instructions (Invitrogen, CA, USA) [32].

### Reverse transcription-quantitative polymerase chain reaction

Total RNA was extracted by Trizol reagent (Sigma-Aldrich, St. Louis, MO, USA) and reversely transcribed by the PrimeScript™ RT Master Mix and SYBR® PrimeScript™ miRNA RT-PCR Kit (TaKaRa, Shiga, Japan). RT-qPCR was performed in the 7500 real-time PCR system (Applied Biosystems Corp., CA, USA) with FastStart Essential DNA Green Master (Roche, Indianapolis, USA) . All primers are listed in Table 1. Glyceraldehyde-3-phosphate dehydrogenase (GAPDH) and U6 were endogenous controls and the 2^−ΔΔCt^ method was applied to calculate gene expression [33].

**Table 1:** Primer sequences for genes used in PCR.

Primer sequences	Forward (5’→3’)	Reverse (5’→3’)
LINC00511	GCACCATCGATCGACCTACA	AAGCAGCTGAGCGAAACTCT
miR-625-5p	AGGGGGAAAGTTCTATAGTCC	Provided in the kit
WEE1	GCTTGCCCTCACAGTGGTATG	CCGAGGTAATCTACCCTGTCTGA
U6	GCTTCGGCAGCACATATACTAAAAT	CGCTTCACGAATTTGCGTGTCAT
GAPDH	GGTCTCCTCTGACTTCAACA	GCCAAATTCGTTGTCATAC

*LINC00511* long non-coding RNA LINC00511, *miR-625-5p* microRNA-625-5p, *GAPDH* glyceraldehyde-3-phosphate dehydrogenase.

### Western blot assay

With tissues and cells lysed with radio-immunoprecipitation assay buffer (Cell Signaling Technology, MA, USA) containing protease inhibitors, protein concentration was measured with a bicinchoninic acid protein assay kit (Beyotime, Shanghai, China). Processed with separation with 10% sodium dodecyl sulfate-polyacrylamide gel electrophoresis, the protein was transferred to a polyvinylidene fluoride membrane and mounted with 5% skim milk. Through incubation with primary antibody WEE1 (1:1000, Abcam, MA, USA) and GAPDH (1:5000, Sigma-Aldrich), the protein membrane was exposed to an appropriate secondary antibody. With GAPDH as an internal control, protein bands were tested on a SupreSignal ECL kit (Pierce, IL, USA) [34].

### Subcellular separation assay

Subcellular separation assay was conducted on a PARIS kit (Invitrogen). RNA distribution (GADPH, LINC00511, and U6) in the nucleus and cytoplasm was tested by RT-qPCR [35].

### FISH assay

The bioinformatics website (http://lncatlas.crg.eu/) predicted the distribution of LINC00511 while FISH assay determined the localization of LINC00511 in SW480 cells. Followed by incubation with a LINC00511 probe (RiboBio, Guangzhou, China), SW480 cells were blocked with 3% bovine serum albumin and rinsed with phosphate-buffered saline/Tween (PBST). Diluted by PBST at 1:800, 4,6-diamino-2-phenylindole (DAPI) was utilized to stain cells. A fluorescence microscope (Olympus, Tokyo, Japan) was applied to capture cell images under five different fields of view [36].

### Dual-luciferase reporter gene assay

A luciferase reporter vector containing the wild-type (WT) sequences of LINC00511 or the 3′-untranslated region (UTR) of WEE1 was constructed. Next, the mutated constructs were constructed following the binding sites. Luciferase reporter vectors were then cotransfected with miR-625-5p mimic or mimc NC. Forty-eight hours post transfection, the relative luciferase activity was counted as the ratio between firefly and Renilla luciferase activities, which was detected by a dual-luciferase system (Promega, Madison, WI). Relative luciferase activity = firefly/Renilla luciferase activity [37].

### RNA-pull down assay

The biotinylated LINC00511 probe was dissolved in 500 µL wash/binding buffer (0.5 mM NaCl, 20 mM Tris-HCL, pH 7.5, and 1 mM ethylenediaminetetraacetic acid). Magnetic beads coated with streptavidin (Life Technologies, Carlsbad, CA, USA) were hatched with the probe and then with cell lysate. Washed twice with cold lysate, three times with low salt buffer, and once with high salt buffer, the RNA complex was eluted from the beads and extracted for RT-qPCR [38].

### MTT assay

After incubation for 24, 48, and 96 h on 96-well plates at 1 × 10^3^ cells/well, cells were added with 5 mg/mL MTT solution at 10 µL/well and continuously incubated for 4 h. After that, cells were supplemented with 100 µL dimethyl sulfoxide and observed for the dissolution of the purple crystals under a microscope. Finally, optical density values were read on a microplate reader (Bio-Tek, Winooski, VT, USA) at 590 nm [39].

### Colony formation assay

The transfected cells were counted on a cell counting plate and fostered in 6-well plates at 0.5 × 10^3^ cells/well for 10 days. The formed cell colonies were fixed with 10% formaldehyde and stained with 0.5% crystal violet solution. After that, the stained cell colonies were photographed and counted under a microscope (Olympus) [39].

### Flow cytometry

Transfected cells were harvested by trypsin, rinsed with pre-cooled phosphate buffer saline, followed by re-suspending in Binding Buffer containing Annexin V-labeled with fluorescein isothiocyanate (FITC) and propidium iodide (PI) (BestBio, Shanghai, China). Upon incubation at 4 °C for 30 min, the apoptotic cells were tested on flow cytometric (BD Biosciences, NJ, USA) [40].

### Scratch test

Transfected cells at 90% confluence in 96-well plates were scraped by a sterile micropipette tip vertically along a ruler. Then, rinsed with PBS, cells were observed for the healing rate at 0 and 48 h with a microscope [36].

### Transwell assay

Transfected cells were seeded into the upper part of the 8-µm Transwell chamber (Corning, NY, USA) coated with Matrigel. The medium (500 µL) containing 15% FBS was added to the lower part. Through incubation under the conventional conditions, the non-invasive cells in the upper part were removed while the invasive cells in the lower part were fixed in 4% paraformaldehyde and stained with 0.1% crystal violet solution, followed by photography under an optical microscope [31].

### Tumor xenografts in nude mice

Nude mice (4–5 weeks old, male) were available from the Model Animal Research Center of Nanjing University (Jiangsu, China) and reared at 25–27 °C with 45-50% humidity. The suspension (1 × 10^6^ cells, 200 mL) of SW480 cells stably transfected with sh-LINC00511 was injected to the left side of the back of mice (*n* = 5). With tumor size measured every 4 days by a vernier caliper, the tumor volume (mm^3^) was calculated as *V* = *A* (length) × *B* (width)^2^/2. After 20 days, the mice were euthanized with CO_2_ to resect tumors. The tumors were photographed and weighed [41].

### Statistical analysis

All data were processed with the SPSS 21.0 statistical software (IBM, NY, USA). The measurement data were expressed as mean ± standard deviation. Except for the discrepancy between tumor tissues and normal tissues evaluated by paired *t* test, the discrepancy between the other two groups was assessed by independent sample *t* test. One-way analysis of variance was adopted to discrepancy among multiple groups, followed by Tukey’s post-test. Repeated measurement of variance was applied to comparisons at different time points with Bonferroni post hoc test. Pearson correlation analysis was used in evaluating the correlation of indicators in clinical samples. Upon *P* < 0.05, statistical significance was constructed.

## Data Availability

The original contributions presented in the study are included in the article/Supplementary Material; further inquiries can be directed to the corresponding author.
